# Medical shift work: a narrative review

**DOI:** 10.47626/1679-4435-2021-881

**Published:** 2023-08-08

**Authors:** Mauricio Vieira Rodrigues, Môsiris Roberto Giovanini Pereira, Daniela Trevisan Monteiro, Paulo Antonio Barros Oliveira

**Affiliations:** 1 Medicina Social, Faculdade de Medicina, Universidade Federal do Rio Grande do Sul, Porto Alegre, RS, Brazil

**Keywords:** shift work schedule, physicians, occupational medicine, jornada de trabalho em turnos, médicos, medicina do trabalho

## Abstract

Shift and night work combine training and practice in medical education, assuming an essential character in some medical specialties. Nevertheless, it is recognized that this work schedule affects biological functions, cognitive performance, and the safety of both patients and workers. The aim of this narrative literature review was to describe current knowledge about the impact of shift and night work in medical professionals. The LILACS, MEDLINE, and SciELO databases were searched for publications between 2010 and 2020 using the terms: “shift work schedule” and “physicians”. A total of 12 publications reported outcomes on sleep quality, family relationships, burnout syndrome, and cardiovascular health. Despite these outcomes, the studies highlighted the importance of shift and night work in medical training, reflecting a loss of learning opportunities when limitations are placed on work hours. The studies suggested initiatives to mitigate the effects of shift work, including increased awareness by managers, a culture of respect for resting periods, the encouragement of family support networks, and the availability of natural light in the workplace.

## Abstract (pt)

O trabalho em turnos e noturno compõe a formação e a prática da educação médica, assumindo caráter essencial em algumas especialidades. Contudo, esse regime de trabalho é reconhecido por gerar efeitos em funções biológicas, na performance cognitiva e na segurança do paciente e do profissional. O objetivo deste trabalho, caracterizado como revisão narrativa de literatura, foi verificar o conhecimento atual sobre o impacto da jornada de trabalho em turnos e noturno na saúde de profissionais médicos. Foram utilizadas as bases de dados Literatura Latino-Americana e do Caribe em Ciências da Saúde, Medical Literature Analysis and Retrieval System Online e Scientific Electronic Library Online, no período compreendido de 2010 a 2020, com os Descritores em Ciências da Saúde: “jornada de trabalho em turnos” e “médicos”. Foram encontrados 82 artigos e, após aplicação dos critérios de inclusão, foram selecionados 12 artigos para análise. Os estudos demonstraram desfechos na qualidade do sono, nas relações familiares, na possibilidade de síndrome de *burnout* e na saúde cardiovascular dos profissionais. Sugerem-se iniciativas para amenizar os efeitos do trabalho em turnos, que incluam a sensibilização de gestores, a cultura de respeito pelo período de repouso, o estímulo à rede de apoio familiar e a oferta de estruturas ambientais de trabalho que permitam acesso à luminosidade natural.

## INTRODUCTION

The aim of shift work, a form of work organization in which teams replace each other at workstations, is to maintain production or service provision in a company or institution for periods > 8 hours per day. Companies or services that use work shifts generally do so for periods ranging from 12 to 24 hours per day and they may or may not continue such scheduling for an entire week, month, or year.^1^

According to Brazilian legislation, night work is activity occurring from 10 pm to at least 5 am the following day.^2^ International Labor Organization Convention 171 defines night work as all work performed over a period of ≥ 7 consecutive hours that includes the period between midnight and 5 am.^3^

In the European Union and North America, between 15 and 30% of workers are employed in some form of shift work.^4^ Another study reported that only 24% of the world’s workforce still has a regular work schedule, ie, Monday to Friday during what are called “traditional” hours.^5^ According to a 2016 nationwide survey by the Brazilian Institute of Geography and Statistics (IBGE), the estimated number of night shift (ie, 10 pm to 5 am) workers was 6.933 million (up from 5.948 million in 2015), totaling 7.6% of the Brazilian workforce.^6^

Due to routine exposure to light during the night and reduced sleep time, shift and night work can act on the central nervous system, disrupting circadian rhythm. Circadian rhythm is an endogenous metabolic process that allows people to predict and anticipate daily environmental changes and adjust behavioral and physiological functions.^7^ Disrupting the circadian system leads to metabolic changes, including the way the autonomic nervous system controls deposition of subcutaneous and abdominal fat, which can result in insulin resistance.^7,8^ Moreover, disturbances in the sleep-wake cycle and in the synchronization of melatonin with circadian rhythm and can result in cognitive dysfunction.^9^

Shift work is also considered a risk factor for several pathologies, including burnout syndrome,^10^ metabolic syndrome, and cardiovascular disease.^8,11^ The link between breast cancer and night work is controversial, with at least two 2019 studies concluding there is no association between them.^12,13^

Health care routinely involves forms of shift work. Studies have investigated the effects of sleep deprivation combined with night shift work, as well as the disruption of homeostatic sleep regulation. In doctors who work the night shift, cognition, psychomotor skills, and surgical performance are affected by sleep deprivation, especially the morning after a shift.^9^ Surgeons report that the physical impact of sleep deprivation can last for days after a night shift. One effect is reduced heart rate variability, which is a measure of physiological stress. In medical residents, sleep deprivation combined with night work have been found to increase the risk of medical error^14^ and traffic accidents when returning home from work.^15^

Workload and sleep deprivation complaints are recurrent in periodic assessments, which is inevitable given the nature of medical work. The compromise to performance and decision-making is well known.^9^ According to the 2020 Medical Demography in Brazil report,^16^ approximately 47% of medical professionals work in shifts and, of these, 62% work 1 or 2 shifts per week, which indicates a higher average weekly workload. Thus, this review is necessary to broaden discussion of the impact of shift work on the daily lives of medical professionals from a pathophysiological and behavioral point of view. Therefore, the general objective of this study was to review current knowledge about the impact of shift and night work on the health of medical professionals, determining which health risks are described in the literature and identifying harmful and/or beneficial aspects of work organization.

## METHODS

This narrative literature review surveyed articles published between 2010 and 2020 in the Latin American and Caribbean Literature in Health Sciences (LILACS), Medical Literature Analysis and Retrieval System Online (MEDLINE), and Scientific Electronic Library Online (SciELO) databases. The search terms were the keywords ‘shift work’ and ‘physicians’ plus the Boolean operator ‘AND’. The searchers were performed between September 2020 and November 2, 2020, resulting in a total of 82 articles (11 from LILACS, 70 from MEDLINE, and 1 from SciELO).

The inclusion criteria were complete texts, published in Spanish, English, or Portuguese, of prevalence studies, clinical trials, qualitative studies, incidence studies, or observational studies. Those that did not focus on shift work by medical professionals, monographs, theses, or dissertations, and studies that addressed only structural aspects of shift work in hospitals without addressing their effects on personnel were excluded. Publications were selected according to PRISMA recommendations.^17^

**Figure 1:**
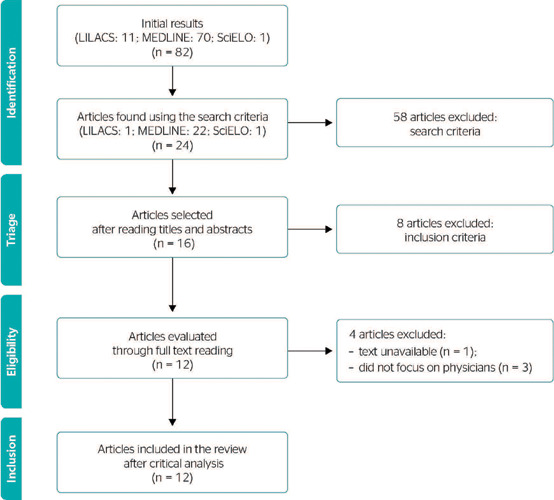
Study flowchart according to Preferred Reporting Items for Systematic Review and Meta-Analysis. LILACS = Literatura Latino-Americana e do Caribe em Ciências da Saúde; MEDLINE = Medical Literature Analysis and Retrieval System Online; SciELO = Scientific Electronic Library Online.

A total of 58 articles were excluded during the selection process, leaving 24 for title and abstract assessment. After an independent reading by 2 evaluators, 8 further studies were excluded. The full texts of the remaining 16 articles were read independently by both evaluators, and 4 more articles were excluded for the following reasons: the full text of 1 was unavailable after repeated searches, and the other 3 investigated the effects of night work on productivity and department structure, but physician health was not a central element. Thus, 12 articles were critically analyzed (Figure 1).

## RESULTS

The purpose of most of the studies was to determine, objectively or subjectively, behavioral and physiological changes resulting from night work. Medical residents were the target population in 9 of the 12 studies. The most common departments were the emergency department, pediatrics, and surgery. The studies were conducted in Brazil, Canada, Chile, France, Japan, Spain, Taiwan, and the USA. Six of the 12 studies used instruments to verify wakefulness and cognitive performance, the most frequent of which were the Epworth Sleepiness Scale,^18,19^ the Psychomotor Vigilance Test, the Karolinska Sleepiness Scale,^20^ and the Maslach Burnout Inventory^21^. These instruments were used in cross-sectional studies to collect prevalence data. One study used a qualitative methodology based on participant interviews.^22^

Four studies used a standard randomized clinical trial format, with interventions ranging from heart rate analysis in groups from different shifts,^23^ measuring sleep-wake activity in recently graduated physicians and residents in intermediate training stages,^20^ the use of text messages to assess sleepiness,^24^ and comparing sleepiness between shift schedules.^25^ Another comparative, nonrandomized study^26^ performed a detailed analysis of physiological, behavioral, and performance variables after a 24-hour shift and in another group of non-sleep deprived personnel. The findings of these studies are shown in Table 1.

**Table 1:** Summary of the literature on medical shift work between 2010 and 2020

Authors	Objectives	Methodology	Results
Francis et al.^27^	To describe the use of sleep-inducing drugs among emergency physicians in a tertiary health care setting in Canada.	Anonymous questionnaire emailed to the clinical staff. Prevalence/cross-sectional study. Descriptive statistics for freguencies and associations between variables.	144 (73%) of 198 physicians responded. 96 (67%) mentioned previous use of sleep aids. 81 (56%) reported current use. Most used nonbenzodiazepines (38%), alcohol (17%), or melatonin (15%); 65 (45%) requested a prescription and 38 (58%) asked a colleague for the prescription. None believed that drug use affected their ability to provide care.
Dutheil et al.^23^	To compare and investigate factors that influence tachycardia and cardiac function among emergency physicians on 24-hour and 14-hour shifts.	RCT comparing HR in 24-h, 14-h shifts, and on a control day. HR was also measured on the third day after shifts.	17 physicians: 100% achieved maximum HR (HR = 180) in both shifts. Min. of tachycardia >100 bpm were higher during a 24 h shift than any other day. Shift days induced twice the cardiac strain of days without patient contact. Each life-and-death emergency induced 26 min of tachycardia (> 100 bpm), 7 minutes (> 110 bpm), 2 minutes (> 120 bpm), and 19 min of cardiac strain ≥ 30%.
Nomura et al.^28^	To explore perceptions of an on-call system to reduce work overload among Japanese residents.	Optional anonymous questionnaire, cross-sectional study (Tokyo NCCHD).	Of 41 residents: 80.4% agreed that patient care quality improved, 26.8% indicated that there was less emphasis on education, 31.7% indicated more emphasis on service, and 90.2% considered the new system beneficial
Lin et al.^18^	To investigate the prevalence of drowsiness and work-related incidents among emergency service health workers (Taiwan).	Online structured guestionnaire based on the ESS. Prevalence/cross-sectional study.	Of 500 workers, 347 provided valid responses: 36.9% indicated mild daytime sleepiness, and 39.2% indicated excessive daytime sleepiness. High ESS scores associated with rotating shifts (increased risk of work accidents).
Morales et al.^26^	To assess, from a multidimensional approach, the stress suffered by medical residents as a result of being on call 24 hours a day (Spain).	Cross-sectional study with 2 groups of resident physicians and selection according to the on-call schedule: 1 (n = 40) sleep-deprived after 24 hours on call; 2 (n = 18) a normal day without sleep deprivation. The following were measured: heart rate variability, Cortisol level, cognitive performance, and mood fluctuations.	Physicians in the 24-hour on-call group had significantly lower mood, performance, and psychological indicators.
Basner et al.^20^	To quantify differences in sleep and wake duration between internists working extended night shifts and residents who rarely or never work extended night shifts.	RCT. Sleep-wake activity in 137 internists and 87 residents (2nd and 3rd year Internal Medicine and Oncology students). Data acquired by wrist actigraph. Wakefulness was assessed with the PVT and KSS.	Interns had fewer hours of sleep (6.93) than residents who did not work extended night shifts (7.18). Lower wakefulness in the mornings after shifts compared to regular shifts. Freguent naps (90.8%) between 9 am and 6 pm on the first day after a shift. Sleep inertia significantly affected alertness in the 60 min after waking on-call.
Nishida et al.^19^	To determine the possibility of brain function deterioration in sleep-deprived residents using neuroimaging technigues.	Randomized crossover study with 6 physicians instructed to collect blood from artificial vessels in a volunteer’s arm. Sleep conditions during the shift were determined by actigraph. The change in cerebral hemodynamics during blood collection was measured with optical topography.	Visual analogue scores after night duty correlated negatively with sleep efficiency during night duty. Reduced right prefrontal cortex activity in the second trial after night duty. The extent of oxygenated hemoglobin decrease in the right dorsolateral prefrontal cortex correlated negatively with ESS score after night duty.
Landmann et al.^29^	To qualify and characterize the nocturnal activity of surgical residents physician.	Prospective 3-phase study of surgical residents during the night shift: needs assessment, direct observation of activities. Oklahoma City, OK, USA.	270 pages were recorded, with 60% defining time-sensitive activities and most being related to pressing patient care issues. Residents spent most of their time (62%) on educational activities. Residents reported overall satisfaction with learning opportunities during the night shift, finding an adequate balance between service and education.
Patterson et al.^24^	To determine: a) the short-term impact of SleepTrackTXT2 on fatigue reported by emergency flight attendants during and at the end of night shifts; b) the long-term impact of SleepTrackTXT2 on sleep quality and sleep health indicators.	4-month RCT of air emergency medical services clinicians. Intervention 1: text messages evaluating sleepiness, fatigue, and concentration at the beginning, every 4 hours, and at end of shifts. Participants who reported symptoms received 1 of 9 behavioral change messages on the night shift. Intervention 2: weekly sleep debt balance report for last 7 days. Control: messages including routine evaluation. Remunerated.	RCT: In 2019, of 83 individuals (2828 shifts) 43 completed follow-up. After 4 months, sleep quality did not differ from baseline within or between groups. Fatigue worsened at 12 h in both groups. Greater fatigue occurred at the end of the shift in controls. < 7 h of pre-shift sleep did not differ between groups. 12-h shifts had a short-term but no long-term impact on fatigue.
Barger et al.^25^	To compare working hours and sleep among resident physicians.	Multicenter RCT. Residents randomized to an EDWR (≥ 24 hours) or a RCWR (≤ 16 hours): 302 medical residents, 370 pediatric intensive care unit shifts at 6 U.S. medical centers (1 month). Sleep estimated by wrist actigraph. Work and sleep hours reported through an electronic diary.	Fewer work hours per week in RCWR than EDWR. 73% of work hours in the RCWR occurred during shifts ≤ 16 consecutive hours. 38% of EDWR work hours occurred in shifts ≤ 16 consecutive hours. Significantly more hours of sleep in RCWR than EDWR. 24-hour intervals with < 4 hours of sleep: 9% in RCWR and 25% in EDWR.
Astudillo et al.^21^	To assess the degree of emotional exhaustion (burnout syndrome), achievement, and depersonalization at work among doctoral students, residents and physicians in the surgery department of a hospital in Chile.	Cross-sectional prevalence study of 19 internists, 11 surgical residents, and 15 surgeons. MBI applied. Measures of central tendency and percentage were calculated; variables compared using Student’s f-test and Cronbach’s alpha coefficients.	Burnout prevalence: 64.4%. Emotional exhaustion dimension: 76%. There was a low rate of work achievement (55%) and a high rate of depersonalization (62%). There were significant differences according to sex, marital status, children, and emergency vs non-emergency shifts. Highest burnout prevalence in: single childless women who work emergency shifts.
Torres & Fischer^22^	To describe time management strategies in the daily routine of internal medicine residents at a teaching hospital in São Paulo.	Qualitative study. Eight interviews conducted with second-year residents about personal and family life, theoretical studies, practical activities, and connection with work. Content analysis with MAXQDA software.	Six thematic categories: “medical residency work organization”, “learning or professional activities”, “financial planning and domestic activities”, “time for leisure and interpersonal relationships”, “family planning” and “rest and sleep routine”.

EDWR = extended duration work roster; ESS = Epworth Sleepiness Scale; HR: heart rate; KSS = Karolinska Sleepiness Scale; MBI = Maslach Burnout Inventory; NCCHD = National Center for Child Health and Development; PVT = Psychomotor Vigilance Test; RCWR = rapidly cycling work roster; RCT: randomized controlled trial.

Generally speaking, the participants acknowledged that night work eventually affects sleep quality,^27^ family relations,^22^ and the likelihood of burnout syndrome.^21^ The studies’ results reveal its impact on physiological, cognitive, and behavioral domains. Most comparative studies^20,23,24,25,26^ investigated physiological and behavioral outcomes according to different shift schedules. Night work affected the heart rate of physicians, including longer periods of tachycardia (> 100 beats per minute)^23^ in doctors who worked 24-hour shifts than those who worked 14-hour shifts, reaching close to 4 hours of tachycardia during a single 24-hour shift. Twice as much cardiac deformation (ratio of shortening and widening of the heart muscle) was observed in those who worked 24-hour shifts. Such cardiac demand is similar to that of tasks involving great physical intensity or exposure to heat and may affect cardiovascular health.^23^

When sleep time was compared between those who worked regular extended night shifts and those who worked sporadic or no night shifts,^20^ a difference of approximately 15 minutes (6.93 hours vs 7.18 hours, respectively) was found. Moreover, it was found that the impact of night work on performance and cognition could be decisive in a moment of emergency. This randomized clinical trial applied the Psychomotor Vigilance Test and Karolinska Sleepiness Scale to assess wakefulness.

Sleep quality was also assessed in a randomized clinical trial^24^ with an intervention group who received text messages tracking drowsiness and signs of fatigue. Physicians who reported these signs received behavioral modification messages during the night shift, which did not prove effective for long-term fatigue control, despite a short-term impact.^24^

One study used wrist actigraphy to estimate the sleep of medical residents, who were randomized between extended shifts (≥ 24 hours) or rapid cycle shifts (≤ 16 hours). It was observed that fewer individuals in the rapid cycle group had 24-hour periods with < 4 hours of sleep.^25^

Another study^26^ found that residents who worked night shifts had lower heart rate variability, increased sympathetic activity, and increased basal cortisol levels, which could lead to a greater risk of cardiovascular disease. Nishida et al.^19^ investigated physicians in 2 sets of conditions: (1) wrist actigraphy to determine the effects of night shift work during the day, prior to an optical topography experiment; (2) home actigraphy applying the Epworth Sleepiness Scale before each phase of the experiment. Lower sleep time and efficiency were found during the night shift than on the day prior to it, but the difference was non-significant (p = 0.345 for both). Optical topography recorded less brain activity in the right prefrontal cortex after a night shift (p = 0.028) while participants drew blood from an artificial vessel.^19^

Questionnaires were a central element in the cross-sectional studies.^18,21,27,28,29^ In one study,^27^ 67% of physicians reported using sleep aids at some point in their careers and that 56% were using them at the time of data collection. The most commonly reported sleep aids were nonbenzodiazepine hypnotics, alcohol, and melatonin, as well as illicit drugs (marijuana) by some participants. A Japanese study^28^ implemented a new on-call system for pediatric residents to improve working conditions, including an 8-hour rest period after the morning team meeting and, after a night shift and case review, residents were relievzed from all patient-related tasks, resulting in approximately 20 hours of rest. In the traditional system, 30 continuous hours were required. The residents reported that care quality improved and fewer adverse effects occurred under the new system, and that their quality of life improved, ie, they felt more motivated and better rested, but they felt the new system was more focused on service than education. Moreover, the results were based solely on subjective perceptions.

A cross-sectional study by Lin et al.^18^ sent online questionnaires (based on the Epworth Sleepiness Scale) to 500 emergency medical service workers in Taiwan regarding workplace sleepiness. A total of 347 workers answered the questionnaire, of whom 281 (81%) reported some type of injury in the last 3 months. This rate increased proportionally with age. Emergency physicians were more frequently unable to work than other medical professionals, which was associated with fatigue and sleepiness due to long working hours. The study’s main finding was the correlation between daytime sleepiness and work disability in emergency room physicians.

Another study^29^ characterized the educational experience that night work provides surgical residents in 3 phases, investigating the balance between educational activities and patient services of no educational value. In the first phase, 29 assessments were received about physician needs, with unanimous approval about the night work experience, characterizing it as an activity with an appropriate level of autonomy. In the second phase, a non-resident surgeon recorded the activity of surgical residents, finding that 104 of the 168 analyzed hours (62%) were dedicated to learning activities, managing critically ill patients, and modifying treatment plans. In the third phase, residents assessed the previous night’s work activities, reporting satisfaction with the balance between education and service in night work. However, most of the surgical residents’ learning time in this shift was not spent in the operating room.

A Chilean study^21^ used the Spanish version of the Maslach Burnout Inventory to evaluate the level of burnout syndrome in 2 surgical services. The questionnaire was applied to 45 participants, including medical interns, surgical residents, and surgeons, of whom 33 (73%) performed shift work. Of the 43 participants who answered the question, 35 (81%) reported sleeping < 7 hours a day. The overall prevalence of burnout syndrome was 64.4%: 79% among medical interns, 63% among residents, and 48% among surgeons. Of the 33 professionals who performed shift work, the overall prevalence of burnout syndrome was 73%, with 27 (82%) reporting emotional fatigue, 21 reporting low professional achievement (63%), and 24 reporting depersonalization (72%).

Finally, Torres & Fischer^22^ sought to describe time management strategies in the routines of internal medicine residents in a São Paulo hospital through a series of 8 interviews that were analyzed qualitatively using content analysis methodology. The results were classified into 6 categories: “work organization”, “learning or professional activity”, “housing, financial planning, and domestic activities”, “time for leisure and interpersonal relationships” “family planning and children” and “rest and sleep”. Regarding rest and sleep time, the residents reported difficulty sleeping, stimulant use, and short naps during the day, which are not effective in terms of rest.

## DISCUSSION

The aim of this review was to survey current knowledge about the impact of shift and night work on the health of medical professionals. Most of the studies reported that shift work, daytime sleepiness, and sleep restriction led to safety risks for both patients and physicians. They primarily focused on the activity of medical residents. Shift work is inherent to medical residency, taking a leading role in some specialties, such as emergency medicine, surgery, and pediatrics.

Due to the prevalence of shift and night work, emergency department personnel are more frequently affected by occupational disease than workers from other specialties. Among these professionals, daytime sleepiness is apparently relevant in the disease process.^30^ The use of sleep aids is rarely reported by medical professionals, usually relegated to studies of residents with a low percentage of respondents. However, even when physicians acknowledge using them, they deny that they impair their ability to provide quality patient care.^27^ Some studies have shown a high turnover of emergency medical service workers due to shift and night work, with some changing their specialty or even their profession.^31^

Medical residency committees have been recognized as a strategy for mitigating the impact of shift work on physician health. For example, in 2003 the U.S. Accreditation Council for Graduate Medical Education^32^ limited the workload of resident physicians to 80 hours of activity per week, with a minimum of 10 hours of rest between shifts and a maximum of 24 hours of continuous activity, with 1 day free of educational and care activities per week. After these restrictions, care failures were reduced by 50% among first-year residents.^33^ However, when surgical residents responded to anonymous questionnaires, they reported that the restrictions curtailed learning opportunities.^34^

In Brazil, Law no. 6,932, of July 7, 1981, in Article 5, provides that medical residency programs will respect a maximum of 60 hours per week, including a maximum of 24 hours on duty, entitled to a weekly day off and a 30-day rest period per year of activity.^35^ Despite the effort to regulate the hours of activity, controversy still exists concerning this hourly limitation in night work, as activities related to night work play an important role in medical training for the management of complex and emergency situations to which physicians will be exposed in their future professional practice.

Although training can restrict sleep time, Baldwin & Daugherty^36^ report that merely reducing medical residency workloads will not necessarily increase sleep duration and may not effectively improve patient safety.^37^ Responding to a questionnaire,^36^ approximately one-quarter of medical residents reported sleeping an average of ≤ 5 hours per night, and two-thirds reported sleeping ≤ 6 hours per night, regardless of household activity. Thus, although the authors concluded that hours of work are related to hours of sleep, this relationship may not be as robust as it seems due to the limitations of retrospective cross-sectional studies (some of which appear in the present review). For example, there could be recall bias regarding hours of sleep, and the quality of sleep (rather than the number of hours) is also determinant, as shown in another publication.^38^

In addition, workload differences in each specialization could lead to different sleeping arrangements, since some residents are can sleep while on call, while others cannot. Nishida et al.^19^ cited this as a limitation, since the sample included residents from a psychiatry unit, whose sleep quality during the shift was comparable to that achieved at home, according to wrist actigraphy. Effective sleep requirements can also vary among individuals, ie, residents with the same workload may have different requirements according to factors such as family and domestic obligations. Financial aspects must also be considered, since trainees may be required to find other remunerated activity to meet the cost of living. Moreover, among established professionals, factors such as family support could mitigate the effects of shift work.^21^

## CONCLUSIONS

Due to the inevitability and unpredictability of disease, health care workers must be prepared to provide patient care at any time. Hence, shift and night work is a necessity in most medical specialties. At some point in their training, all physicians perform learning or professional tasks during shift or night work. Measures to regulate work hours and restrict extended shifts were consolidated at the beginning of the 21st century. However, these restrictions, especially those regarding professional training, have been subsequently questioned by the beneficiaries themselves despite acknowledging better quality of life they provide. Moreover, shift work tends to be incorporated into the routine of trained professionals and involves long-term consequences, such as sleep aid use, cardiovascular problems, and burnout syndrome.

More comprehensive research will require the participation of a larger number of professionals and adherence to follow-up, which in some studies was incipient. There is implicit concern about data collection issues, such as accuracy in reporting of hours of sleep (which is limited in retrospective approaches), and recurrent use of psychotropic drugs. In addition, most of the reviewed studies only involved medical residents, and shift work is a relevant part of professional training programs. Hence, studies of fully trained professionals would to improve analysis of the impact of shift work. Initiatives to mitigate the effects of shift and night work are needed, including awareness raising among both managers and physicians, encouraging a culture of respect for rest periods (ie, avoiding overlapping work activities that restrict sleep time), stimulating family support networks, and, whenever possible, providing work environments with natural light, which contributes to chronobiological synchrony.
